# High fat diet significantly changed the global gene expression profile involved in hepatic drug metabolism and pharmacokinetic system in mice

**DOI:** 10.1186/s12986-020-00456-w

**Published:** 2020-05-24

**Authors:** Yuqi He, Tao Yang, Yimei Du, Lin Qin, Feifei Ma, Zunping Wu, Hua Ling, Li Yang, Zhengtao Wang, Qingdi Zhou, Guangbo Ge, Yanliu Lu

**Affiliations:** 1 The Key Laboratory of the Minstry of Education of the Basic Pharmacology and the Joint International Research Laboratory of Ethnomedicine of the Ministry of Education, School of Pharmacy, Zunyi Medical University, 6 West Xue-Fu Road, Zunyi City, 563009 Guizhou China; 2 Institute of Chinese Materia Medica, Shanghai Key Laboratory of Complex Prescription and the Ministry of Education (MOE) Key Laboratory for Standardization of Chinese Medicines , Shanghai University of Traditional Chinese Medicine, Shanghai, China; 3 School of Pharmacy, Philadelphia College of Osteopathic Medicine, Suwanee, GA USA; 4 School of Chemistry, The University of Sydney, Camperdown, NSW2006 Australia; 5 Institute of Interdisciplinary Integrative Medicine Research, Shanghai University of Traditional Chinese Medicine, Shanghai, China

**Keywords:** High fat diet, Drug metabolism and pharmacokinetic, RNA-Seq, Transcriptome

## Abstract

**Background:**

High fat diet impact transcription of hepatic genes responsible for drug metabolism and pharmacokinetics. Until now, researches just focused on a couple specific genes without a global profile showing. Age-dependent manner was also not noted well. This study aims to investigate the high fat diet effect on transcriptome of drug metabolism and pharmacokinetic system in mouse livers and show the age-dependent evidence.

**Methods:**

C57BL/6 male mice were used in this experiment. High fat diet was used to treat mice for 16 and 38 weeks. Serum total cholesterol, low density lipoprotein cholesterol, aspartate transaminase, and alanine transaminaselevels were measured. Meanwhile, Histology, RNA-Seq, RT-PCR analysis and fourteen major hepatic bile acids quantification were performed for the liver tissues. Data was mined at levels of genes, drug metabolism and pharmacokinetic sysem, and genome wide.

**Results:**

Treatment with high fat diet for 38 weeks significantly increased levels of serum lipids as well as aspartate transaminase, and alanine transaminase. Meanwhile, lipid accumulation in livers was observed. At week 38 of the experiment, the profile of 612 genes involved in drug metabolism and pharmacokinetics was significantly changed, indicated by a heatmap visulization and a principal component analysis. In total 210 genes were significantly regulated. Cyp3a11, Cyp4a10, and Cyp4a14 were down-regulated by 10–35 folds, while these three genes also were highly expressed in the liver. High fat diet regulated 11% of genome-wide gene while 30% of genes involved in the hepatic drug metabolism and pharmacokinetic system. Genes, including *Adh4, Aldh1b1, Cyp3a11, Cyp4a10, Cyp8b1, Fmo2, Gsta3, Nat8f1, Slc22a7, Slco1a4, Sult5a1,* and *Ugt1a9*, were regulated by high fat diet as an aging-dependent manner. Bile acids homeostasis, in which many genes related to metabolism and transportation were enriched, was also changed by high fat diet with an aging-dependet manner. Expression of genes in drug metabolism and disposition system significantly correlated to serum lipid profiles, and frequently correlated with each other.

**Conclusions:**

High fat diet changed the global transcription profile of hepatic drug metabolism and pharmacokinetic system with a age-dependent manner.

## Background

Precision medicine has the potential to change the practice of medicine by tailoring treatments to individuals. By giving a patient with the right drug at the right dosage with a right form at a right time, the health care could be delivered with greater accuracy and efficiency, leading to better health outcomes. In addition to how molecules affect targets in human bodies, how human bodies dispose xenobiotics is also important for precise therapeutic effect. Once a molecule enters our biology system, it may go through various metabolic process including oxidation, hydrolysis, methylation, glucuronidation, etc. Hundreds of enzymes and transporters participate in these processes. As we know until now, the drug metabolism and pharmacokinetic (DMPK) system includes a large number of families such as cytochrome P450 (CYP), flavin-containing monooxygenase (FMO), monoamine oxygenases (MAO), esterases, UDP-glucuronosyltransferases (UGT), methyltransferases (MET), APT binding cassettes (ABC), and so on [1]. In different human body status, such as physiological and various pathological conditions, the expression levels of these aforementioned proteins are changedaffected by the status of physiological and various pathological conditions, and thenleading to the alterations of the drug absorption, metabolism, transportation, and eventually the pharmacokinetic profiles are altered. Thus, if the expression levels of these enzymes and transporters changed, the medication dosages and even the drug delivery forms may have to be adjusted accordingly. Understanding the change in DMPK system in various pathological statuses is the key step to guarantee the precision of the therapeutic effect.

Diets are one of the most important ways that humans and animals exchange substances and energies with the environment. A lot of diseases are caused by abnormal and unhealthy diets. Among those unhealthy diets, high fat diets are reported to be associated with various diseases such as fatty liver diseases, diabetes, hyperlipidemia, hyperglycemia, atherosclerosis, and even neuron system diseases. In the very beginning, high fat diet was a label of traditional western food. However, with the economy development, high fat diets become more popular in China, Korea, Japan, and other eastern Asian countries where vegetables and carbohydrates were the traditional foods. Most of these diseases are associated with functions of livers which is the most important organ responsible for drug metabolism and disposition. If the liver function changed, the expression levels of genes or proteins in the DMPK system are altered accordingly. In vivo pharmacokinetic profile, such as bioavailability, half life, and even tissue distribution, of medicines treating these diseases would become abnormal. Finally, the dosage and drug delivery system would be re-designed to promise the effecacy and safety. Thus, understanding the effect of high fat diets on DMPK system is essential to guarantee the therapeutic precision.

The impairment on a couple DMPK genes expression of caused by high fat diet has been reported in several studies. Long term treatment of high fat diet significantly reduced the mRNA levels of *Cyp2a4*, *Cyp2b10*, and *Cyp3a11*, as well as their regulators such as nuclear receptors CAR, PXR, and RXR2. In the fatty liver diseases rat model, gene expression levels of *Cyp1a2*, *2c11* and *3a2* were strongly reduced [2]. High fat and glucose diet induced rat hepatic gene expression levels of *Ugt1a6*. In high fat diet rat model, protein expression levels of CYP2E1 and CYP4A were elevated [3], which could be caused by the expression and activation of nuclear receptors CAR and PPARα [3]. In both patients and murine models with non-alcohol fatty liver diseases (NAFLD), *Cyp4a14* is up-regulated, while *Cyp4a14* seems to be a therapeutic target of NAFLD [4]. However, for another fatty lipid catabolism enzyme *Cyp4a10*, high fat diet significantly repressed its gene expression [5]. In patients with non-alcohol steatohepatitis (NASH), the serum arachidonic acid homeostasis mediated by cytochrome P450 enzymes was completely dysregulated [6]. *Cyp7a1*, *Cyp8b1*, and *Cyp27a1*, three important bile acids biosynthesis enzymes, were up-regulated in patients with NAFLD [7]. In addition, transporters such as NTCP (Slc10a1), OATP1B1, and OATP1B3, were also up-regulated. Abnormal gene expression levels of CYPs was not not only found in livers, but also in other tissues. For example, gene expression levels of *Cyp2j5*, *Cyp2j6*, and *Cyp2c44* in adipose tissues were reduced by high fat diet [8]. Accompanied with the effect on expression and activation of CAR and PPARα, UGT1A1 and UGT1A6 expression levels were altered by the high fat diet [3]. Regarding transporters, gene expression level of *Abcc3* was reduced by high fat diet [9].

Although data showed so many DMPK members regulated by high fat diet, there are still some unsolved problems. For example, a research regarding high fat diet and drug metabolizing enzymes revealed that high fat diet regulates endogenous CYPs in a gene-specific manner [10]. Until now, hundreds of CYP isozymes have been identified, but the effect of High fat diet effect on CYPs has not been fully understood. On the other hand, other than CYPs, the effect of high fat diet on other phase I metabolizing enzymes such as MAO, NAT, esterases, etc. is still not understood. Regarding phase II enzymes and transporters, only a few of them were investigated. We conducted a comprehensive literature and database searchto collect more than 600 DMPK genes, unfortunately, the understanding of the high fat diet effect on most of those genes as well as on the global DMPK system is still defficient.

Thus, in the present study, with a RNA-seq technology, we investigated the transcriptome of mouse hepatic DMPK system to profile the high fat diet induced changes. In addtion, we also investigated age-dependent impatct of high fat diet on a couple representative genes involved in this system. We hope findings of the present study would help the understanding of high fat diet effect on drug metabolism and disposition and help the clinical study of the same topic.

## Methods

### Materials

Male C57BL/6 mice (23–25 g) were purchased from Huafu Kang Biological Technology Co., Ltd. (Beijing, China; approval number: SCXK 2014–0004). Standard chow diet (23.2% of calories from protein, 12.1% from fat, and 64.7% from carbohydrate) was purchased from Jiangsu Xietong Biomedical Engineering Co., Ltd. (Jiangsu, China). High fat diet (20.0% of calories from protein, 60.0% from fat, and 20.0% from carbohydrate) was purchased from Research Diets Inc. (New Brunswick, USA). Total cholesterol assay kit, Low-density lipoprotein cholesterol assay kit, Aspartate aminotransferase Assay Kit, and Alanine aminotransferase Assay Kit were purchased from Nanjing Jiangcheng Bioengineering Institute (Nanjing, China). DEPC water was purchased from Zoman Biotechnology Co., Ltd. (Beijing, China). Bile acid was purchased from Sigma-Aldrich Co., Ltd. (St. Louis, Missouri, United States). Ammonium acetate was purchased from Thermo Fisher Scientific Co., Ltd. (Waltham, Massachusetts, USA). Acetonitrile and methanol were purchased from Tedia Co., Ltd. (Ohio, United States).

### Animal experiments

Male C57BL/6 mice were kept in the well-controlled animal room on the SPF (Specific Pathogen Free) level. Room temperature was set to 21–23 °C, while the humidity adjustment ranged from 50 to 60% and a 12 h light/dark cycle was used. Animals were free to ingest food and water, monitored body weight trends every day. In this study, we involved 55 mice in total and set three time points including day 0, week 16, and week 38. At the start of the experiment, 11 mice in group day-0 were sacrificed without any treatment. The other 44 mice were divided into 4 groups (*n* = 11 per group), treated by normal diet (ND) or high fat diet (HFD) for 16 or 38 weeks. At the end point of each group, mice were anesthetized by 7% chloral hydrate (0.005 mL/g) and blood was taken from the eyeball. After incubating at room temperature for 1 h to obtain serum, blood was centrifuged at 3500 rpm for 10 min to obtain serum. A slice of liver samples was fixed in 10% formaldehyde solution, while rest of the livers and other tissues were placed in liquid nitrogen for quick-freezing, and then stored in − 80 °C refrigerator. All mice were in accordance with the requirements of Animal Experiment Ethics Committee of Zunyi Medical University.

### Histology analysis

The HE staining (hematoxylin and eosin staining) was used to analyze histopathology of liver tissues. The liver tissues were fixed in 10% formalin solution for 24 h, dehydrated with alcohol and placed in xylene. The fixed liver tissues was embedded in the paraffin (Leica EG1150, Wetzlar, Germany) and then cut into slices of 6–8 μm by freezing microtome (Leica RM2245, Wetzlar, Germany). Tissue slices were washed, dehydrated by ethanol, and stained by hematoxylin and eosin for Olympus BX43 microscopic examination (Tokyo, Japan).

### Assays of serum cholesterol, glucose, ALT, and AST levels

Serum total cholesterol (TC), low density lipoprotein cholesterol (LDL-C), aspartate transaminase (AST), and alanine transaminase (ALT) of mouse treated by normal diet or high fat diet for 16 or 38 weeks were assayed according to the manufacturer’s instructions and results obtained by microplate spectrophotometer. The blood glucose levels were measured by using ROCHE Accu-Chek Performa.

### RNA preparation and sequencing

For each group, 5 out of 11 mice livers were randomly picked for RNA sequencing. Total RNA was extracted from mouse liver tissues according to following procedures. Approximately 20 mg of liver tissues was homogenized in 1 mL Trizol on ice, incubated at room temperature for 5 min and added 200 μL chloroform. After low temperature centrifugation, 500 μL isopropanol was used to precipitate RNA. The precipitate was washed with 75% ethanol (DEPC water configuration), and then dissolved by 30 μL DEPC water after evaporated all ethanol. RNA integrity was evaluated by Agilent 2100 Bioanalyzer (Agilent Technologies). RNA sequencing was performed on an Illumina Hiseq 2000 platform (BGI, the Beijing Genomics Institute, https:// www.genomics.org.cn).

### The cDNA synthesis and quantitative real time PCR

Double-stranded complementary DNA (cDNA) was synthesized from total RNA by using a PrimeScript RT reagent kit (Company, City, TaKaRa, Japan) on a Mastercycler Gradient PCR Thermal cycler (Eppendorf Scientific, Inc., Germany), according to the manufacturer’s instructions. Quantitative Real-time PCR was performed by using 2*SYBR Green Supermix (Bio-Rad, Germany). The RT-qPCR reactions were conducted on a CFX96 RT-PCR System (C1000 Touch, BioRad, Germany). Transcriptomic data were validated by performing qRT-PCR on *Cyp7a1* and *Cyp8b1* to detect its mRNA levels at four groups (ND16W, HFD16W, ND38W and HFD38W). The expression of GAPDH was used as the normalization control. The primers used were listed in Table 1. The Pearson product-moment correlation coefficient (R) was used to evaluate the strength of association between qRT-PCR and RNA-seq results.

**Table 1:** Primers for real time PCR assays

Gene	Forward primers	Reverse primers
*Cyp7a1*	GAGCCCTGAAGCAATGAAAG	GCTGTCCGGATATTCAAGGA
*Cyp8b1*	GGACAGCCTATCCTTGGTGA	GACGGAACTTCCTGAACAGC
Cyp4a10	AATGGGAACGGCTGGCTGA	TGGCCAAGCGGCCATTGGAA
Cyp4a14	GGCGAAAGAAAGTCAGGTT	AGGCAGTGTTCAGTTGGA
Cyp4a31	AATGGGAACGGCTGGCTGGT	TGGACAAGCGGCCATTGGA
Cyp2c70	TGGAGCCGGAACAGAGACAACGA	ACGGCATGTGGTTCCTGTCCTGCA
Cyp3a59	CAGGCTAGAAAGGGTCTCAAAA	AGTTTCATGCTGATGAGAGCAA
Gsta3	AGGAGTGGCGGATCTGGAGA	CGGGTCCAGCTCTTCCACAT
Gstm6	GCCATGGTTTGCAGGGGACA	TGGAAGGAAGCGGCTGGTCT
Gstm7	CCATGGTTCGCAGGGGACAA	TTGCTGCCCCAAGTTGCCA
Nat8f1	ACAGCATCGAGGACAGGGGA	GCCTGCCTTCTTGAAGCCCA
Slc22a30	GGAAGTATTATTTGTCTCCCATCG	AGGATCCTACTGCCATTATCAGAG
Slc22a5	ACGAAGCCTCAGTTGCACCA	ACAACAGCCAGGCCAGCACA
Slc30a10	GACTTTCATTTGCTGTGCTGAG	GTGACAGGACATAAGGCAATGA
Slc4a4	CTTACAGAATCCCCAAGACTGG	AAGTTGCATAGGAAAAGGGACA
Slco1a4	TACACGCTGGGTCGGTGCTT	TCGGATTGCAGGAGAGGCT
Ugt2b35	GCCCTTGCCCAGATTCCACA	AGAGTCCATTCGCGCCACCA
Cyp3a11	TGCCTTGGCATGAGGTTTGC	TGACTGCATCCCGTGGCACA
Cyp2c70	TGGAGCCGGAACAGAGACAACGA	ACGGCATGTGGTTCCTGTCCTGCA
*Gapdh*	TGTGTCCGTCGTGGATCTGA	CCTGCTTCACCACCTTCTTGA

### Bile acids quantification

Hepatic bile acids homeostasis profiles of all mice involved in the animal experiment were checked by the ultra performance liquid chromatography coupled with tandom mass spectrometry (UPLC-MS/MS) technology. Approximately 100 mg of liver tissues was homogenized in 300 μL acetonitrile on ice. The homogenates were centrifuged at 14300 rpm and 4 °C for 10 min. Take the supernatant 250 μL, which was evaporated under nitrogen gas flow nitrogen blowing dry and dissolved in 100 μL 50% methanol. Quantifications of BAs were performed on a 6420 Triple Quad UPLC-MS/MS system (Agilent 6420, Santa Clara, CA). An CQUITY UPLC BEH C18 (1.7 μm, 100 × 2.1 mm) column was used for BAs separation, eluting with acetonitrile and ammonium acetate as the mobile phase. The chromatographic parameters were refered to the published method [11]. Parameters of the ESI ion source were as follows: Gas Temp: 326 °C, Gas Flow: 12 L/min, Nebulizer: 55 psi, Capillary: 3500 V. The mass analyzer was set as the SIM mode to capture the [M-H] ^−^ ions of expected BAs. Peak areas of each observed BAs were used for comparison and statistical analysis among groups.

### Data process and mining

**Fig. 1:**
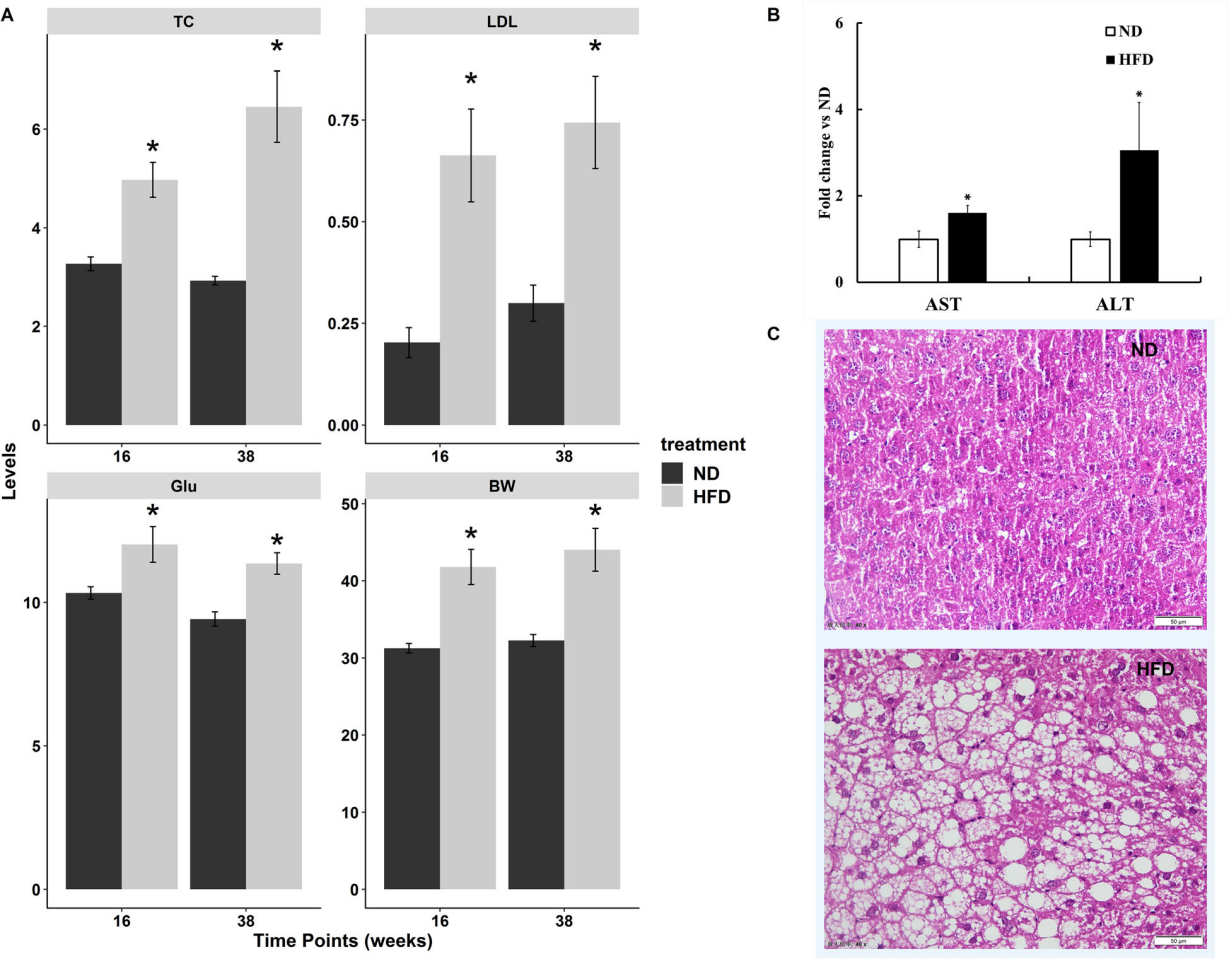
High fat diet changed lipid profiles in serum and liver tissues at week 38. **a** High fat diet significantly increased the serum total cholesterol (TC), low density lipoprotein cholesterol (LDL), blood glucose (Glu), and body weight (BW) levels of mice at both week 16 and 38. **b** High fat diet significantly increased the serum AST and ALT levels of mice at week 38. **c** Liver histopathology (HE, × 40) showing the fat accumulation in hepatocytes of mice treated by high fat diet for 38 weeks. Abbreviation: ND, normal diet group; HFD, high fat diet group. Data were presented as mean ± SEM. ^*^p < 0.05 checked by the two-way ANOVA forllowed by the Tukey’s test

**Fig. 2:**
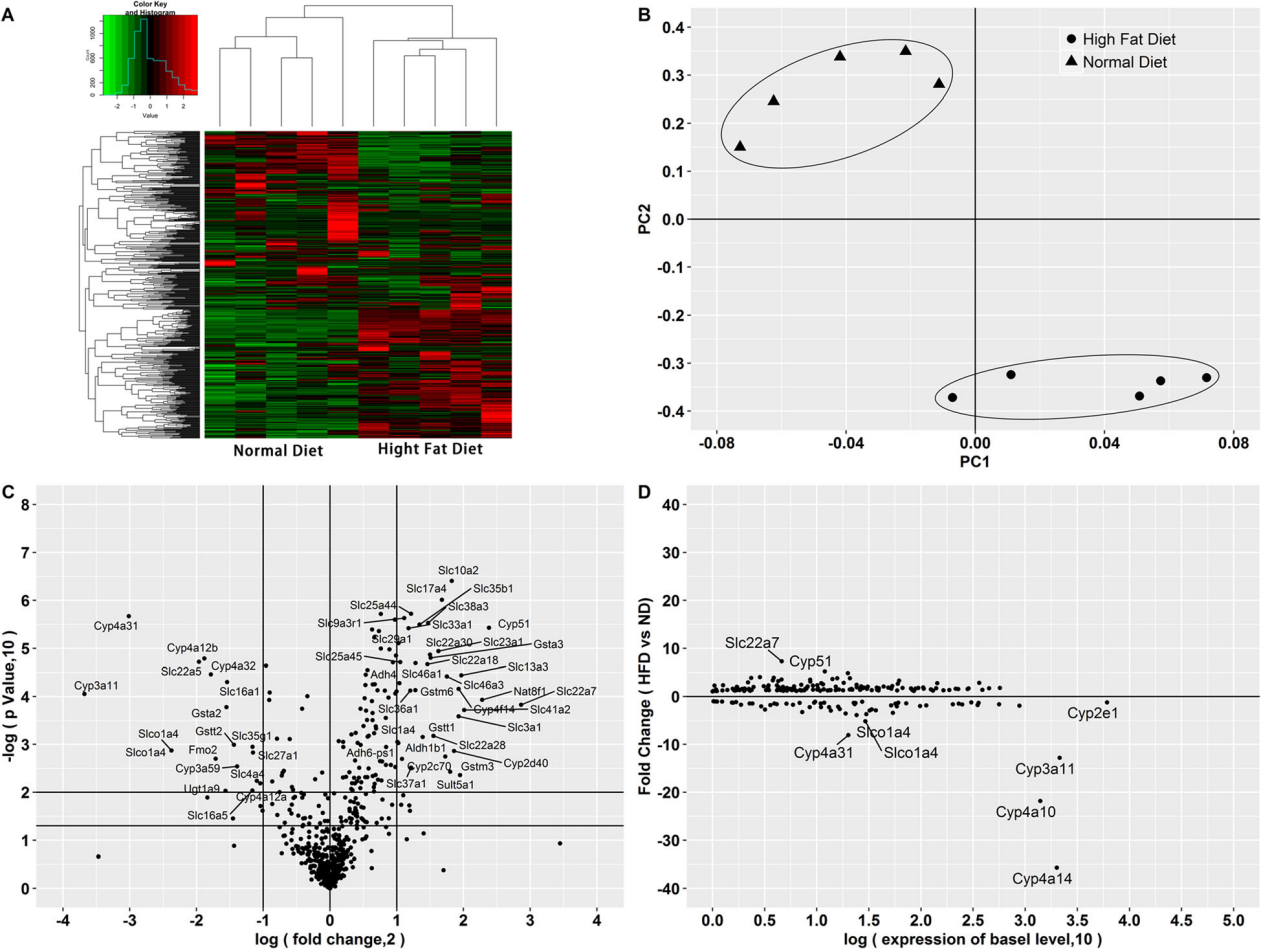
Expression profiles of hepatic DMPK genes changed by treatment of high fat diet for 38 weeks. **a** Heatmap visualization. **b** Score plots of principal component analysis. **c** Volcano plots showing the significantly regulated genes. Names of genes changed by more than 2 folds with *p* < 0.01 were labled. The top and bottom horizontal line represent *p* values of 0.01 and 0.05, respectively; **d** Distribution of the expression levels of genes significantly changed by treatment of high fat diet for 38 weeks. Names of genes with fold change values more than 5 or with basel expression levels (FPKM) more than 1000 were labeled. Abbreviation: ND, normal diet group; HFD, high fat diet group

**Fig. 3:**
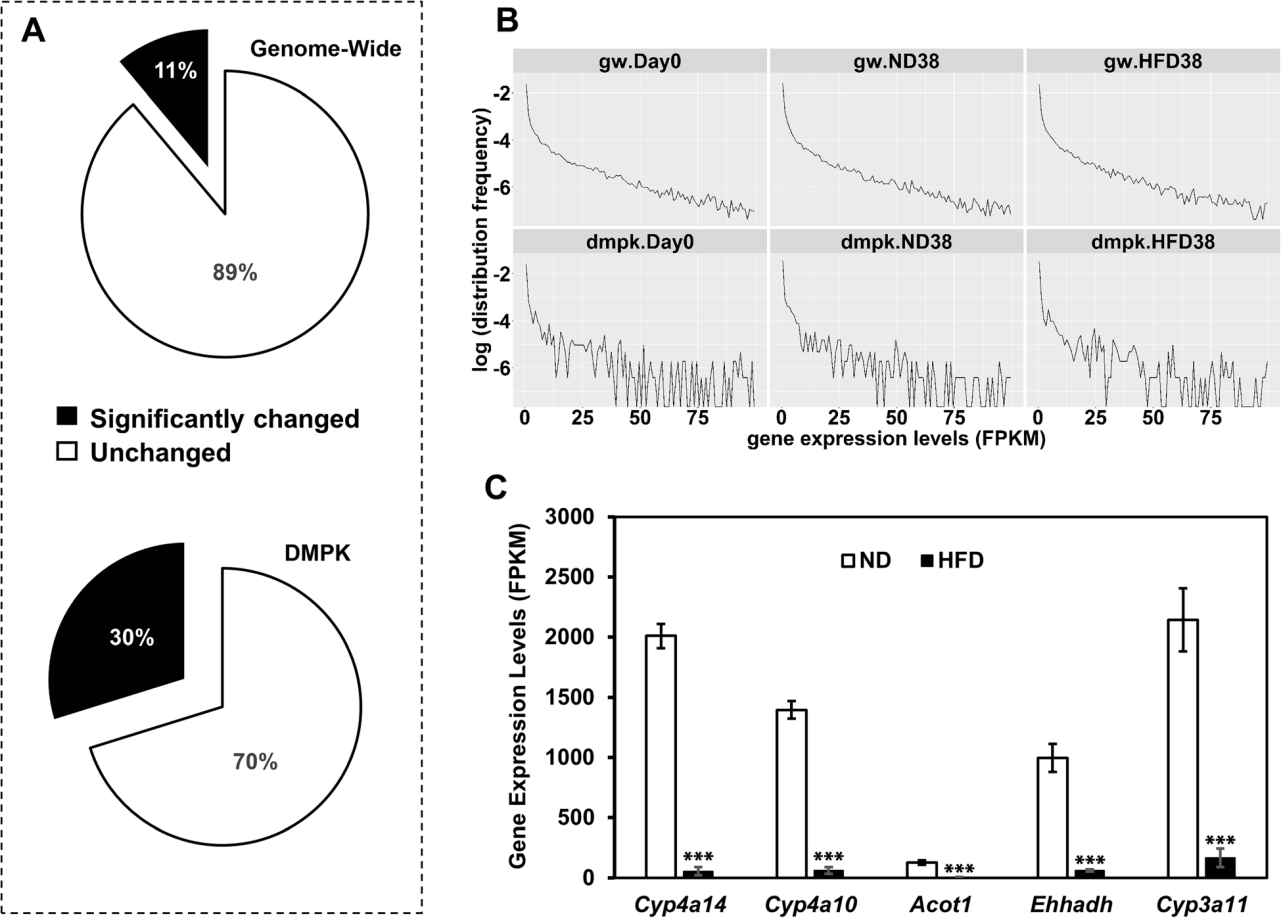
Effect of high fat diet on DMPK system play an important role on its effect on the whole genome. **a** The percentage of genes significantly changed by high fat diet at the transcriptional level. Top: whole genome wide scale; bottom: DMPK system. **b** Distribution frequency of hepatic gene expression levels at the whole genome wide (gw) scale and DMPK system scale. Mice were treated for 0 day (day 0) or 38 weeks. . **c** Top five HFD-repressed genes on the genome wide levelsData were presented as mean ± SEM. ^***^p < 0.001 checked by the student t-test. ND, normal diet group; HFD, high fat diet group

Variance analyses and significance tests were performed using SPSS Statistics 18.0 (IBM, Chicago, USA). Statistic methods were described in the figure legends. In the present study, we generated a list of approximately 600 genes involved in DMPK systems, and this gene set has been reported in NCBI database (https://www.ncbi.nlm.nih.gov/). R program was used to visulize the expression levels of all investigated DMPK gene expressions and profile the global changes [12]. The heatmap was generated by using the “heatmap.2” function of the gplots package in the R program [13]. Other figures were prepared by the “ggplot” function of the “ggplot2” package in the R program [14]. Principal component analysis (PCA) was performed using the “pca” function of mixOmics package in the R program [15]. Before principal component analysis, data was scaled based on gene. Components number was set to be 2. Statistical significance levels of data in Figs. 1, 5, and 6 were tesed by the two-way ANOVA followed by a Tukey’s test, which were done by using the base functions “aov” and “TukeyHSD” in R program. In Fig. 2c and Fig. 3c, the statistical significance levels of data were tested by the student t’test, which was done by using the base function “t.test” in R program.

## Results

### High fat diet changed lipid profiles in serum and liver tissues

Treatment with high fat diet for both 16 and 38 weeks significantly increased serum total cholesterol level (Fig. 1a), and low-density lipoprotein cholesterol level by two-fold. In addition, blood glucose levels and body weights were also induced. Meanwhile, hepatic injury was present by the elevated levels of serum AST and ALT (Fig. 1b). The HE staining showed an obvious accumulation of fat in liver cells (Fig. 1c). All of those data indicated that high fat diet made the lipids homeostasis disordered and may accomany with liver injury.

### Hepatic profiles of genes enriched in the hepatic DMPK system

In the present study, we generated a list with genes involved in DMPK systems. In total 612 genes were collected. Based on this gene set, hepatic expression profiles of mice treated with high fat diet for 38 weeks were obviously different from that treated with normal diet. In heatmap (Fig. 2a), genes located in lower side showed the similar expression pattern. These genes had higher hepatic expression levels in mice treated with high fat diet. In contrast, genes located in upper side showed similar expression patters that mice treated with normal diet had higher gene expression levels than high fat diet. A principle component analysis (PCA) showed completely different location between mice treated with normal diet and high fat diet in score plots (Fig. 2b), indicating that expression profiles of gene transcriptions involved in DMPK systems were significantly affected by high fat diet. Based on these gene expression data (Fig. 2c), we generated a volcano plot showing how these genes were changed by high fat diet. Among all 612 genes, hepatic expression levels of 210 genes were significantly altered. Among these 210 significantly regulated genes, 64 genes were down regulated, while 146 genes were up regulated. If we considered the fold change of the gene expression levels, 24 genes were down-regulated by twice, while 39 genes were up-regulated by twice.

To observe the favored expression level region regulated by high fat diet, we used the data of 210 genes which were significantly regulated by treatment of high fat diet for 38 weeks. We plotted the fold change values vs the basel expression levels of these 210 genes. The basel levels of these 210 genes distributed from FPKM value of 0 to more than approximat 10,000. Genes encoding a couple major cytochrome P450 enzymes responsible for most xenobiotic metabolism, like *Cyp3a11* and *Cyp2e1*, showed relatively high expression levels in mice livers (Fig. 2d). The genes of *Cyp4a10* and *Cyp4a14*, which are responsible for fat metabolisms, were also highly expressed in mice livers. Interestingly, most of these top expressed DMPK genes were significantly affected by high fat diet. For example, gene expression level of *Cyp4a14* was down regulated by more than 35 times. Gene expression of *Cyp3a11*, an important xenobiotic metabolic enzyme, was down regulated by more than 10 times. In contrast, although a large amount of DMPK genes were significantly regulated (Fig. 2c), genes with lower expression levels were not regulated as sensitive as highly expressed *Cyp3a11*, *Cyp4a10*, and *Cyp4a14* (Fig. 2d). Regarding genes with FPKM values less than 1000, only *Slc22a7*, *Slco1a4, Slc22a7, and Cyp51* were regulated by more than 5-fold.

### Significantly regulated hepatic genes were enriched in DMPK systems

Although more than 20,000 hepatic genes were detectable by RNA-sequencing, most of them were expressed in the livers at extremely low levels. The majority of hepatic genes accumulated in the area with expression values (FPKM) less than 100 (Fig. 3b). We assume that most of those genes are silenced in physiological condition. Regarding genome wide genes with FPKM values more than 100 (*n* = 679), high fat diet treatment for 38 weeks regulated 11% of them **(**Fig. 3a**,** top pan chart**)**. However, if we changed the scale to DMPK system, the percentage values increased to 30% (Fig. 3a, bottom pan chart). To observe the enriched area of expression levels for genes regulated by high fat diet in both genome wide and DMPK scale, we plotted the distribution frequency of gene number against the gene expression levels represented by FPKM values. High fat diet did not make a signicant change in both genome wide and DMPK system scale (Fig. 3b). However, in the top 5 genes regulated by high fat diet on the genome wide scale (in total 20,521 genes), 3 out of them, including *Cyp4a10*, *Cyp3a11*, and *Cyp4a14*, belong to the hepatic DMPK system **(**Fig. 3c**),** proving again that DMPK system is an import area in the liver suffering from the pressure of high fat diet.

### RT-PCR validation of a couple selected genes

**Fig. 4:**
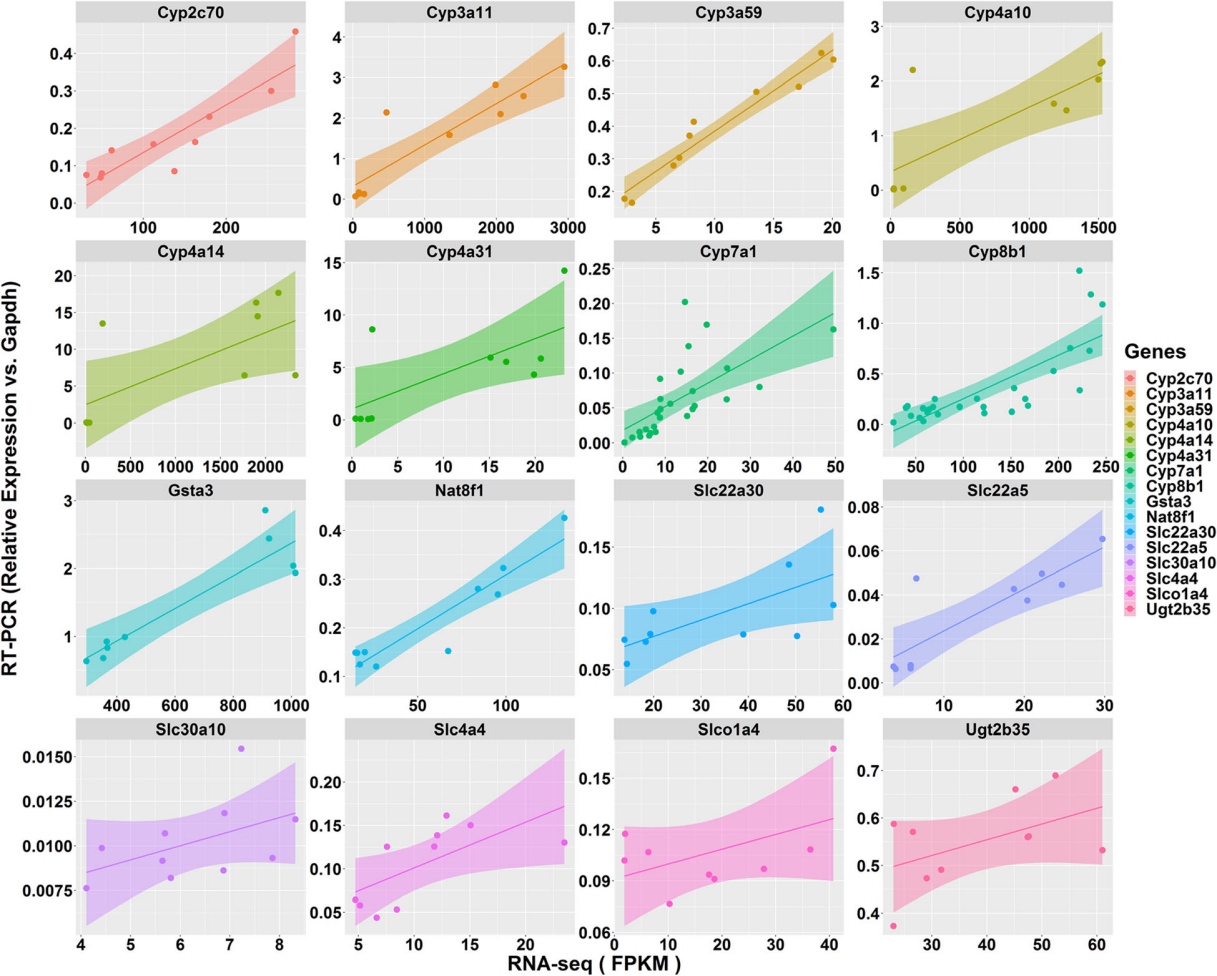
Validation of RNA-seq data by real-time PCR technology on 12 randomly selected genes involved in the drug metabolism and pharmacokinetic system. Shadows means the confidential area of the trend lines

Although the accuracy and precision of mRNA chips and RNA sequencing were well accepted. In this study, we still randomly picked a couple genes involved in hepatic DMPK system to check the expression levels by traditional real-time PCR. The real-time PCR data and the RNA-seq data referring to the same sample was compared (Fig. 4). RNA seq data of all of the investigated 16 genes showed a positive relationship with the data accquired based on traditional real-time PCR technology. However, we also observed that, the correlation between two technology is closer in high expression area than low expression areas, indicating one of the technologies may more sensitive that the other one. As the real-time PCR highly depend on the primer sequence, we assume the RNA seq data is more reliable. Any way, this comparison proved that the analysis could be checked by more than one technology.

### Effect of high fat diet on DMPK genes in mice with different ages

**Fig. 5:**
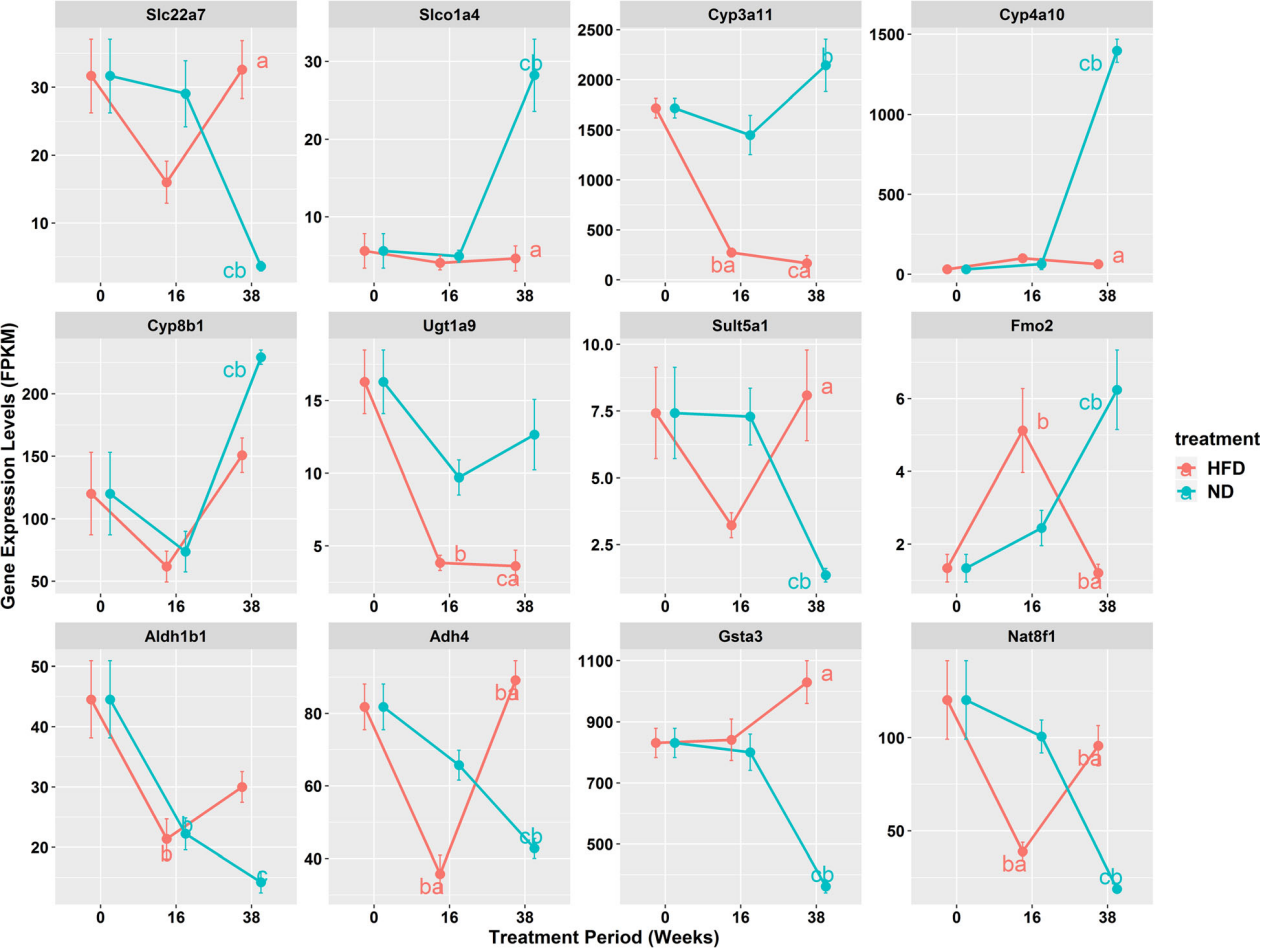
Time course of hepatic expression levels of 12 represented DMPK genes in mice treated by normal diet or high fat diet for 0 week, 16 weeks and 38 weeks. Abbreviation: ND, normal diet group; HFD, high fat diet group. Data were presented as mean ± SEM. ^a^*p* < 0.05 between ND and HFD groups at a specified time point. ^b^*p* < 0.05 vs the previous time point with the same treatment. ^c^*p* < 0.05 vs the week 0. All comparison were checked by the two-way ANOVA followed by the Tukey’s test

In the present study, mice serum total cholesterol, low density lipoprotein cholesterol, and blood glucose levels were significantly induced at week 38. At this time point, mice were relatively old. As we started the experiment while mice were 8 weeks old. At week 38 of the experiment, the mice were already 46 weeks old and could be used as an aging model. To observe the gene regulation profile by high fat diet in younger mice, we set the other time point of week 16. Among all significantly regulated DMPK genes at week 38, we picked 12 of them to test. The 12 genes consist of members from CYP, UGT, FMO, NAT, GST, SLC, SULT, ADH, and ALDH families, covering the most DMPK functions. Proteins encoded by these 12 genes were responsible for xenobiotic oxidation, conjugation, as well as transportation. Almost all of them were regulated by high fat diet at a age-dependent pattern **(**Fig. 5**)**. Some of them, like *Cyp3a11* and *Ugt1a9*, were constitutively repressed by high fat diet treatment for both 16 weeks and 38 weeks. The fold changes that high fat diet repressed them at week 38 were apparently larger than that at week 16, however, fail to pass the statistic chack. Some of them, like *Slc22a7*, *Adh4*, and *Nat8f1*, were repressed by high fat diet at week 16, however, were induced at week 38. Moreover genes involving Slco*1a4*, *Cyp4a10*, *Cyp8b1*, *Aldh1b1*, and *Gsta3*, were not regulated by high fat diet at week 16, however were significantly induced or repressed at week 38.

### High fat diet altered bile acids homeostasis in which lots of DMPK genes were enriched

**Fig. 6:**
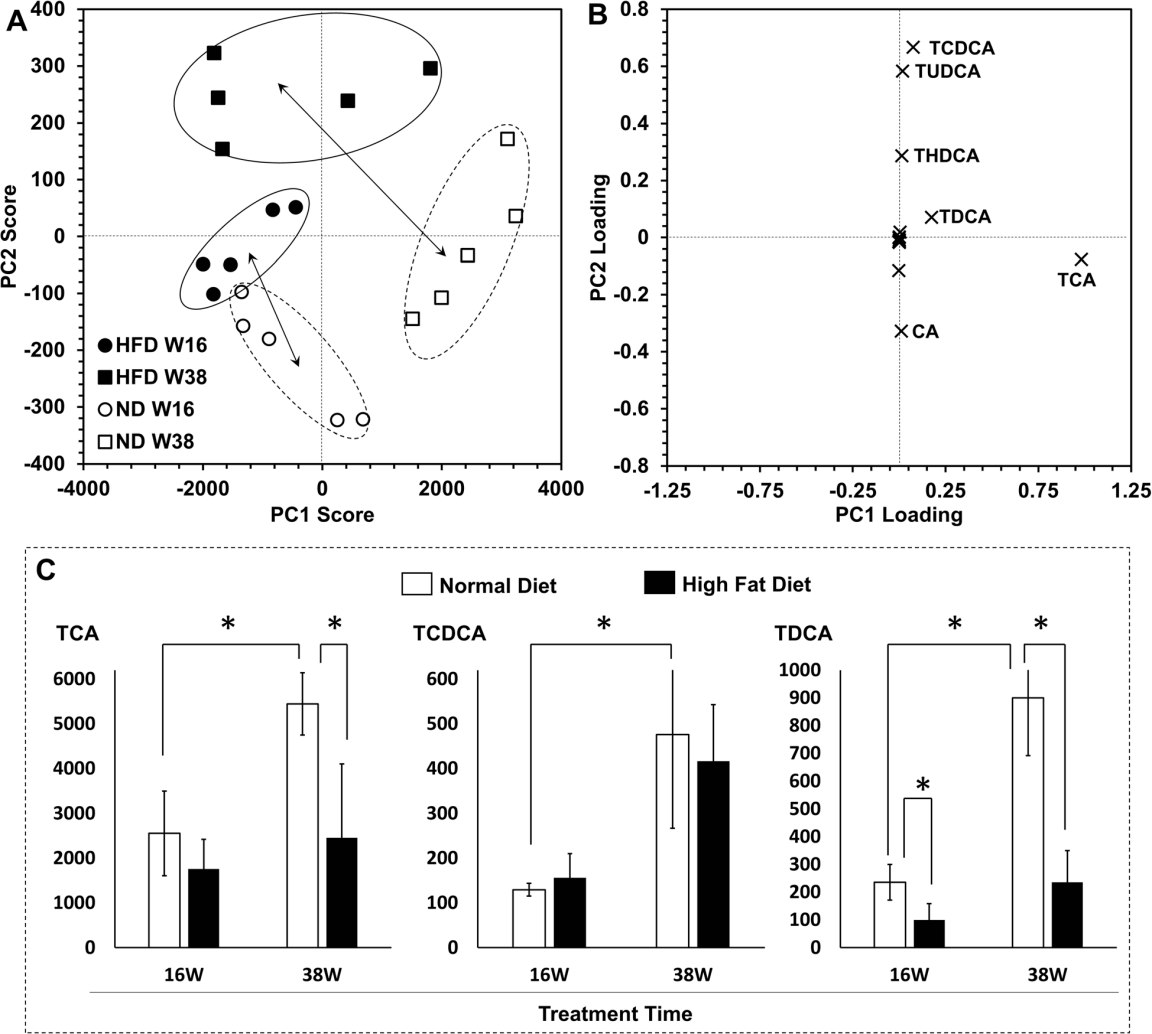
Effect of high fat diet on the bile acids pathway. **a** Score plot of principal component analysis. **b** Loading plot of principal component analysis. **c** TCA, TCDCA,TDCA levels. Abbreviation: ND, normal diet group; HFD, high fat diet group. Data were presented as mean ± SEM. ^*^p < 0.05 checked by tne-way ANOVA followed by the Tukey’s test

Mouse hepatic bile acids levels were determined by ultra performance liquid chromatography coupled with tandem mass spectrometry. Fourteen major hepatic bile acids were quantified in mice treated by normal diet and high fat diet for 16 and 38 weeks. Score plots of principle component analysis clearly distinguished mice received different treatments, as well as mice at different ages **(**Fig. 6a**)**. It indicated that high fat diet significantly changed hepatic bile acids homeostasis in both young and old mice. The distance between old mice treated by high fat diet and normal diet in score plots was obvious farer than that between young mice treated by high fat diet and young mice (**Indicated by the arrows in** Fig. 6a). It showed hepatic bile acids homeostasis in old mice is more sensitive to high fat diet than in young mice. Loading plots suggested TCA as a significant marker contributing to mice treated with normal diet for 38 weeks (Fig. 6b). One-way ANOVA verified the statistic significance of the higher TCA level in old mice treated with normal diet in contrast to old mice treated with high fat diet, as well as to young mice treated with normal diet (Fig. 6c). However, high fat diet had no effect on TCA levels in young mice. Bile acid TCDCA was not changed by high fat diet in both young and old mice livers, meanwhile, TDCA was altered by both high fat diet treatment and aging.

### Correlation analysis on the DMPK systematic level

**Fig. 7:**
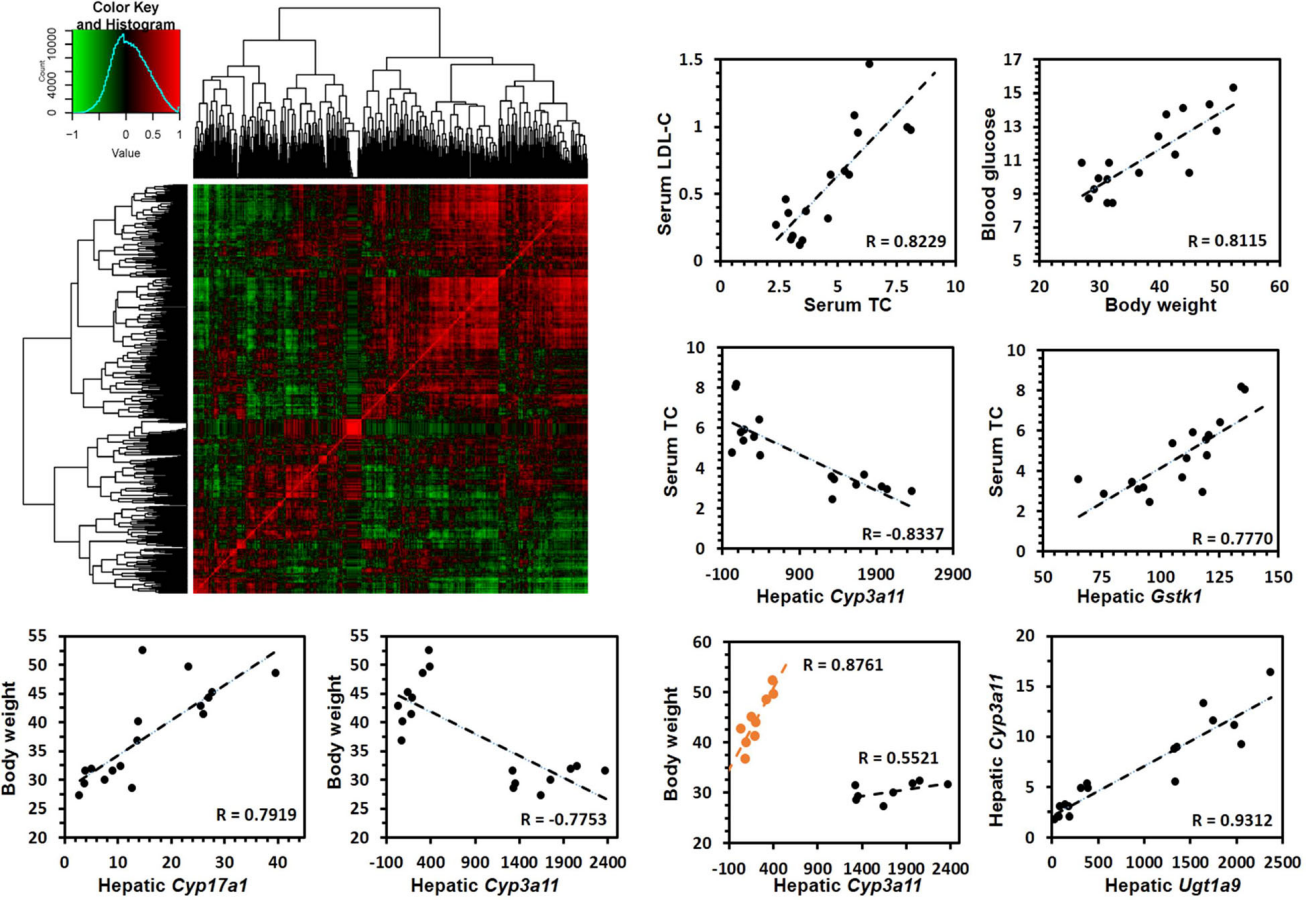
Correlation between serum lipids profile and DMPK gene expressions. Heatmap showed correlation between expression levels of genes involved in drug metabolism and pharmacokinetic system. Line plots showed the correlation between expression levels of representative genes and serum lipids as well as between each other of specific genes

Using gene expression levels and serum lipid levels in mice treated with normal diet and high fat diet for both 16 and 38 weeks, a correlation analysis was conducted. A heatmap showed that large numbers of genes were ether positively (indicated by the red color, Fig. 7) or negatively (green color) correlated with each other. Serum total cholesterol levels were positively correlated with serum LDL-C levels. Body weight was positively correlated with blood glucose. These findings were consistent with traditional opinions. Some were also identified from the correlation analysis. For example, serum total cholesterol levels were negatively correlated with hepatic gene expression levels of *Cyp3a11*, however, positively correlated with that of *Gstk1*. Mice body weight was positively correlated with hepatic gene expression levels of *Cyp17a1*, while negatively correlated with that of *Cyp3a11*. Interestingly, transcription of Cyp3a11 was repressed by high fat diet and showed opposite trend with the mouse body weight, however, within the high diet group or normal diet group, Cyp3a11 gene expression levels were unexpectedly positively

correlated with body weight. In addition to the relationship between body weight as well as serum lipid profiles and gene expressions, correlation between genes were also observed. For example, *Cyp3a11* gene expression was positively correlated with *Ugt1a9* in liver.

## Discussion

As the deficiency of global analysis of gene expression profile of hepatic DMPK systems, the effect of high fat diet on a lot of important DMPK genes was still not known. In the present study, we filled some unknown areas. *Ugt1a9* encodes an enzyme responsible for hydroxyl and phenol glucuronidation. Substrates of UGT1A9 involve lots of nature products such as flavonoids, tanshinone, and some alkaloids [16–19], as well as important synthetic drugs such as propofol [20], 4-methylumbelliferone [21], mycophenolic acid [22], and morphine [23]. In addition, UGT1A9 also participated in metabolism processes of endogenous signaling molecules like estradiol [24]. In the present study, we showed that *Ugt1a9* was down regulated by high fat diet by more than 3 folds. As drugs like propofol and mycophenolic acid are extremely important for surgery anesthesia and organ transplantation, respectively, dosages should be adjusted when patients have long term of high fat diet history. Except *Ugt1a9*, in the present study, we found around 170 high fat diet regulated genes which have not been reported before, including frequently studied CYP and UGT families. For example, we observed a 5-folds down-regulation of *Fmo2* by high fat diet in the present study. FMO family members are responsible for catalysis of oxidation of nitrogen atom which is included in structures of lots of drugs. In addition, *Fmo2* is an important source of hydrogen peroxide, thus is important for xenobiotic-induced toxicity [25]. Anti-tuberculosis drug ethionamide is mainly metabolized by FMO. As ethionamide may induce acute liver injury, reduced gene expression of *Fmo2* may associate with high safety issue if the dosage was not adjusted in patients with high fat diet history. Moreover, recent years, more peptide drugs have been developed [26]. In the present study, we also observed a couple high fat diet regulated genes responsible for peptides metabolisms such as *Nat8f1*, *Nat8f2*, *Mgst3*, and *Gstm6*. Metabolisms of peptide drugs are completely different from traditional small molecular drugs. CYP and UGT enzymes almost do not participate in the metabolism processes of peptide drugs [27]. Nitrogen acetylation as well as peptide conjugations may play much important roles. Nitrogen terminal acetylation may improve the stability of peptides [28]. We observed a 5-folds up-regulation of *Nat8f1* by high fat diet in this study. Thus, we assume that increasing the dosage of peptides drugs in patients with high fat diet history is a potentially valuable topic in clinical researches.

Different from traditional views, the biological functions like DMPK are not determined by a couple specific genes but a global system or even global systems. Many nutritional statues,such as starvation and fasting, could altere the pharmacokinetic characteristics of drugs, among which high fat diet was the most common situation used in the experiment process [29]. In this study, we have analyzed the transcriptome of mouse hepatic DMPK system including in total 612 genes. The results showed that mice treated with high fat or normal diet displayed a different gene expression profile in a global view. Among those 612 DMPK-involded genes, based on the RNA-seq data, we have identified 146 up-regulated and 64 down-regulated genes in high fat diet group as compared to normal diet group, supporting the hypothesis that high fat diet changed the DMPK system profile but not only a couple specific genes.

As we were discussing the change of DMPK system by high fat diet at the gene expression levels. We also want to know some sign on the functional level. Regarding DMPK, structure changes and transportation processes are very important. In vitro, we could use some probes to check. However, even though the in vivo probe exists for a couple DMPK enzymes, defficence of probes for most enzymes and transporters is still the problem. Thus, we have to find a indirect way. The best way is the usage of a endogenous metabolic network. In a certain period following drug administration, the concentrations of a drug and its metabolites in the circulating system and target organs were controlled by drug metabolism and transport in organs, such as liver, intestine and so on [30]. The liver is the major organ for drug metabolic processes, including phase I and II. In addition, large and charged compounds are normally transported into the hepatocytes and out into the bile by drug transporters [31]. Enzymes and transporters participating in these aforementiond processes are also frequently enriched in the system controlling the bile acids homeostasis. Although it is a known knowledge that high fat diet changed the bile acids pool and profiles, the data coming from the same sample sets maybe more accurate for comparison. Results of the present study as well as the reported work did support our hypothesis. Bile acids homeostasis was significantly changed by high fat diet. More important, same as the gene expression profile, the change of bile acids homeostasis by high fat diet also followed the age-dependent manner.

*Cyp4a14* is an important gene coding the protein for lipid oxidation. To data, Cyp4a14 was up regulated by the high fat diet treatment in 26 weeks. However, 38 weeks after high fat diet treatment, we observed a dramatically down-regulation of this gene. It implies that the regulation of DMPK genes may be time-dependent or age-dependent. This is a very important finding indicating the different regulation style of high fat diet on DMPK system may exist in elder people from young people. On the other hand, high-fat diet increases fatty acid oxidation levels in mice [32]. As the high levels of serum lipids still existed at week 38 of high fat diet treatment, how to release the block of the transcription process of lipid oxidation genes like Cyp4a14 is the potential direction of drug development. Regarding *Cyp2e1*, to data, this gene was up-regulated in mice treated by a high fat diet of 8 weeks [33]. In the present study, this gene was also induced by a high fat diet treatment of 16 weeks, however, down-regulated of 38 weeks. *Cyp2e1* enzyme expresses in many tissues including liver and other organs. It has an important role in oxidizing various small molecule substrates such as alcohol, drugs, solvents and fatty acids [34]. In addition, *Cyp2e1* regulation is affected by age, gender, genetic factors, nutrition, hormones, and pathophysiological conditions such as diabetes and obesity. The reports that *Cyp2e1* levels are regulated during aging, which was consistent with our result [35].

CYP7A1 is a rate-limiting enzyme that converts cholesterol into bile acids in the liver, can contribute to maintenance of mammalian cholesterol homeostasis [36]. High fat diet treatment of 8 weeks repressed mRNA level and protein level of *Cyp7a1* in rat [37], consistent with our result. However, in patient with NAFLD, the trend is opposite [7]. Species difference may exist between rodents and humans.

In HFD/STZ-induced diabetes mice model, mRNA level of *Cyp8b1* was induced [38]. However, a high fat diet treatment unique showed a down-regulation trend on *Cyp8b1* [33]. *Cyp8b*1 was a key enzyme in the bile acid formation pathway. Our study shows that high fat diet mice were inhibited expression of *Cyp8b1* and *Cyp7a1*. Cholesterol catabolism into bile acids was inhibited, which caused the cholesterol elevated cholesterol [39].

*Cyp3a11* is a mouse homolog of *Cyp3a4*. It has been reported that hepatic *Cyp3a11* expression decreased after high fat diet feeding in mice. This is in line with previous reports11. The strong inverse correlation between RNA expression of *Cyp3a11* and serum TC or body weight implicate that high fat diet may directly reduced RNA levels of the hepatic *Cyp3a11* enzymes compared to normal diet mice. *Cyp3a11* participates in the metabolisms of hypnotic drug midazolam. The prolonged sleep time induced by the experimental midazolam in high fat diet mice indicates a decrease in the expression of *Cyp3a11*, demonstrating that the presence of a high-fat diet is indeed reflected in its pharmacodynamics [40]. Therefore, dosages should be adjusted when patients have long term of high fat diet history taking hypnotics. *Cyp17a1* has 17,20-lyase activity, which determines the final product of steroid hormone biosynthesis. Studies have reported that 17,20-lyase activity leads to androgen, whereas 17-hydroxylase activity of *Cyp17a1* is necessary to produce glucocorticoid cortisol [41]. The positive correlation of seen *Cyp17a1* gene and body weight in the present study may imply that *Cyp17a1* can contribute to the development of metabolic syndrome. In addition, Our studies showed that high fat diet mice, the down-regulation genes included *Cyp8b1*, *Cyp7a1*, *sult3a1*, *Sulte1*, *Gstt3*, *Cyp51*, *Cyp2c54* and *Cyp4f14*, and the up-regulation genes included *Cyp17a1*, *Cyp3a41a*, *Asns*, *Past1*, *Cyp2c55*, *Gstm2* and *Gstaα1*. Studies suggested that besides *Cyp17a1*, *Cyp3a41a* were opposite, others consistent with literature [33].

A similar study has investigated the high fat diet effect on rats hepatic DMPK system at the transcriptional level [42]. In a 12-weeks treatment by using high fat diet, a couple mice hepatic phase I and phase II metabolic enzymes as well as some transporters were down-regulated at the transcriptional level. In the present study, we found a consistent trend in a 16-weeks treatment by high fat diet on genes such as *Cyp1a2, Cyp3a11 (Cyp3a1/2 in rats), Cyp4a12, Ugt1a1, Ugt1a6, Ugt1a9, Ugt2b1, and Gstt1*. However, at week 16, high fat diet did not regulate the expression levels of mice hepatic genes *Slco1a2, Slco1b2, Slc22a5, Abcc2, Abcc3, Abcb1a, and Abcg2* which were down-regulated in the reported 12-weeks treatment of rats by high fat diet. As some important genes showed the same trends in our study as the reported data, the significant role of high fat diet on the regulation of DMPK system was confirmed again. As the experiment parameters were not completely matched between the present study and the rat experiments, the different regulation style of some other genes was acceptable. At first, species are diffent. A couple DMPK genes in mice and rats are not the same as each other. Secondly, mice were treated by high fat diet one more week in the present study than rats in the aforementioned paper. Finaly, we used normal diet as the control but low-fat diet was used as the control in rats experiment. However, both researches indicated that species differnce was worth to study on the effect of high fat diet on DMPK system.

## Conclusions

High fat diet changed the global profile of hepatic gene transcription of the drug metabolism and pharmacokinetic system but not only a couple specific genes involved. The impact of high fat diet on drug metabolism system was more serious than on the whole genome. The effect of high fat diet on the effect of high fat diet on DMPK gene transcription showed an age-dependent or feeding-duration-dependent style. It is worthable to watch the medicine dosage in patients with high fat diet history in the futher clinical researches.

## Data Availability

The datasets used and/or analysed during the current study are available from the corresponding author on reasonable request.

## Abbreviations

HFD: High fat diet
ND: Normal diet
TC: Total cholesterol
LDL-C: Low density lipoprotein cholesterol
AST: Aspartate transaminase
ALT: Alanine transaminase
